# Airway Epithelium Dysfunction in Cystic Fibrosis and COPD

**DOI:** 10.1155/2018/1309746

**Published:** 2018-04-08

**Authors:** Virginia De Rose, Kevin Molloy, Sophie Gohy, Charles Pilette, Catherine M. Greene

**Affiliations:** ^1^Department of Clinical and Biological Sciences, University of Torino, A.O.U. S. Luigi Gonzaga, Regione Gonzole 10, 10043 Orbassano, Torino, Italy; ^2^Department of Medicine, Royal College of Surgeons in Ireland, Education and Research Centre, Beaumont Hospital, Dublin 9, Dublin, Ireland; ^3^Institute of Experimental and Clinical Research, Pole of Pneumology, ENT and Dermatology, Université Catholique de Louvain (UCL), Brussels, Belgium; ^4^Department of Pneumology, Cliniques Universitaires St-Luc, Brussels, Belgium; ^5^Lung Biology Group, Department of Clinical Microbiology, Royal College of Surgeons in Ireland, Education and Research Centre, Beaumont Hospital, Dublin 9, Dublin, Ireland

## Abstract

Cystic fibrosis is a genetic disease caused by mutations in the CFTR gene, whereas chronic obstructive pulmonary disease (COPD) is mainly caused by environmental factors (mostly cigarette smoking) on a genetically susceptible background. Although the etiology and pathogenesis of these diseases are different, both are associated with progressive airflow obstruction, airway neutrophilic inflammation, and recurrent exacerbations, suggesting common mechanisms. The airway epithelium plays a crucial role in maintaining normal airway functions. Major molecular and morphologic changes occur in the airway epithelium in both CF and COPD, and growing evidence suggests that airway epithelial dysfunction is involved in disease initiation and progression in both diseases. Structural and functional abnormalities in both airway and alveolar epithelium have a relevant impact on alteration of host defences, immune/inflammatory response, and the repair process leading to progressive lung damage and impaired lung function. In this review, we address the evidence for a critical role of dysfunctional airway epithelial cells in chronic airway inflammation and remodelling in CF and COPD, highlighting the common mechanisms involved in the epithelial dysfunction as well as the similarities and differences of the two diseases.

## 1. Introduction

**Figure 1:**
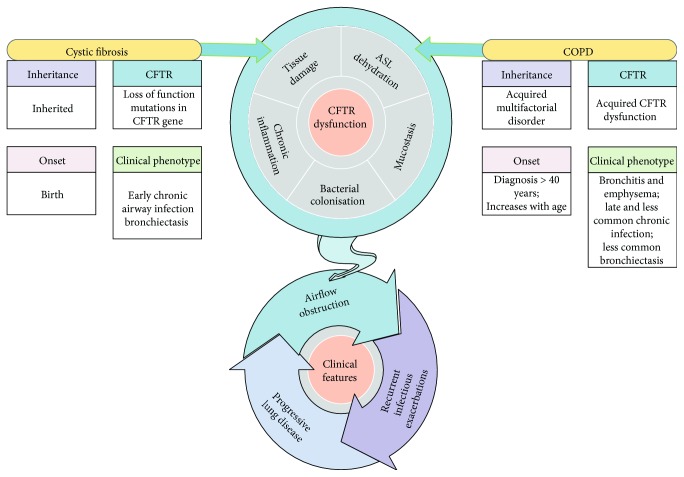
Similarities and differences between cystic fibrosis and COPD. Cystic fibrosis (CF) is a genetic disease caused by mutations in the CFTR gene, while COPD is a multifactorial-acquired disease of later onset predominantly associated with cigarette smoke exposure. Although the diseases are different in many aspects, they also share key phenotypical features. Recent evidence suggests that cigarette smoke induces an acquired CFTR dysfunction in COPD and that both CF and COPD have corresponding molecular phenotypes of CFTR dysfunction resulting in airway surface liquid (ASL) dehydration, mucostasis, bacterial colonization, chronic inflammation, and tissue damage. Airflow obstruction, recurrent infective exacerbations, and a progressive decline in lung function characterize both conditions.

Cystic fibrosis (CF) is the most common genetic disease in the white population and results from mutations in a single gene encoding for a 1480 residue transmembrane glycoprotein, the cystic fibrosis transmembrane conductance regulator (CFTR). Lung disease, characterized by chronic neutrophilic inflammation, progressive airflow obstruction, and airway bacterial infections, is the major cause of morbidity and mortality in patients with CF [1, 2]. Chronic obstructive pulmonary disease (COPD) is a major global health problem, and it is estimated to become the third leading cause of death worldwide by 2020; the disease is caused by both genetic and environmental factors; among these, cigarette smoking is the main risk factor for COPD and triggers an inflammatory response throughout the airways, in the alveoli, and in the pulmonary vasculature [3, 4]. The two predominant phenotypes in COPD are emphysema and chronic bronchitis; however, they often overlap in most patients. Although CF and COPD are different in many aspects, they also share key phenotypical and pathologic features suggesting potential mechanistic links (Figure 1). Both diseases are associated with progressive airflow obstruction, chronic neutrophilic inflammation in the airway lumen, and recurrent infectious exacerbations; disease exacerbations significantly contribute to morbidity and mortality in both diseases and have a relevant impact on patient's quality of life as well as on health care expenditures [1–4].

Airway epithelial cells (AEC) are among the first sites of contact for inhaled insults and play a crucial role in maintaining normal airway function [5, 6]. The airway epithelium represents the primary site of relevant molecular and histologic changes in both CF and COPD, and growing evidence in recent years has suggested that it plays a key role in disease initiation and progression in both diseases. Structural and functional changes in both airway and alveolar epithelium have a relevant impact on alteration of the airway milieu, host defences, and the repair processes that contribute to the airflow limitation characteristic of both diseases. The airway epithelium could therefore represent a suitable target for novel therapeutic strategies aiming to restore barrier integrity and defences against inhaled particles and pathogens. In this review, we address the evidence for a critical role of dysfunctional airway epithelium in impaired local defences, altered immune responses, chronic airway inflammation, and remodelling in CF and COPD, highlighting the common mechanisms involved in epithelial dysfunction as well as the similarities and differences of the two diseases.

## 2. Properties of Airway Epithelium

The airway epithelium acts as a physical barrier to prevent potential pathogens or noxious agents entering airway mucosa and reaching the bloodstream [7]. In addition, the airway epithelium controls ion transport to keep the airways hydrated and, furthermore, acts as a key regulator of innate immune responses toward invading pathogens.

### 2.1. Barrier Function

The crossing of one or multiple host barriers by a pathogen is critical to initiate infection. The airway epithelium provides a physical barrier to microbial invasion; it consists of a monolayer of polarised epithelial cells which maintain their integrity by apical junction complexes (AJC). These consist of occluding tight junctions (TJ), anchoring adherens junctions (AJ), desmosomes, and GAP junctions.

TJs regulate the movement of ions, macromolecules, and immune cells through the paracellular space; they are critical for ion transport and act as a barrier to regulate the access of inflammatory cells and against the entry of harmful substances such as microbial components into the airway lumen. TJs are constructed from zona occludens proteins (ZO), occludin, claudins, and junctional adhesion molecules (JAMs). AJs mediate cell–cell adhesion and signalling pathways that control cell growth, morphology, and differentiation. They are located below TJs in the lateral membrane and utilise cadherins to mediate calcium-dependent cellular adhesion by binding a cadherin of the same type on an adjacent cell [8]. Gap junctions are not part of AJCs and are pathways of intercellular communication constructed with channel proteins called connexins [9].

### 2.2. Mucociliary Escalator

The airway is maintained in a constant state of hydration through the coordinated actions of the CFTR channel and amiloride-sensitive epithelial sodium channel (ENaC). The airway surface liquid layer (ASL) comprises a mucus layer which functions to trap particulate matter, bacteria, and viruses, and the underlying periciliary liquid layer (PCL), which provides hydration, enabling effective mucociliary transport and clearance [10]. A finely tuned balance between chloride secretion by CFTR on the one hand and sodium absorption by ENaC on the other keeps the ASL sufficiently hydrated to permit effective and sustained mucus clearance via the mucociliary escalator; additional ion channels contribute to maintain ASL homeostasis (reviewed in Section 3). Mucus dehydration, defective mucociliary clearance of microbes, infection, and inflammation are the hallmarks of CF and COPD lung disease.

### 2.3. Pathogen Sensing

**Figure 2:**
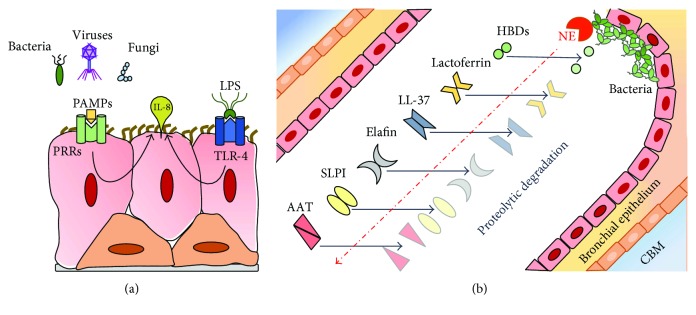
Airway innate immunity. (a) The major pattern recognition receptor (PRR) is the Toll-like receptor (TLR) family which interact with microbial-derived factors or pathogen-associated molecular patterns (PAMPs) such as LPS from *P. aeruginosa*. LPS-induced activation of TLR induces an IL-8 response via a NF-*κ*B pathway. (b) Important antiproteases and antimicrobial proteins in the lung include AAT, SLPI, elafin, the cathelicidin LL-37, lactoferrin, and the human beta defensins (HBDs); these can be cleaved and inactivated by neutrophil elastase in the CF lung. CBM: capillary basement membrane.

The airway epithelium senses pathogens using pattern recognition receptors (PRRs) including Toll-like receptors (TLRs), RIG-I-like receptors (RLRs), Nod-like receptors (NLRs), C-type lectin receptors (CLRs), protease-activated receptors (PARs), and the bitter and sweet taste receptors, amongst others. TLRs are expressed by epithelial cells throughout the respiratory tract including tracheal, bronchial, and alveolar type II cells and respond rapidly to infection or tissue damage by sensing local microbial and host-derived factors (Figure 2(a)). The nucleic acid-sensing TLRs (TLR3, TLR7, and TLR9) and the RLRs, RIG-I, and MDA-5 provide defence against viruses invading the respiratory tract [11], by generating IL-6, CXCL8 (IL-8), and IFN-*β* responses. RIG-I and MDA-5 expression is increased in influenza A virus-infected nasal epithelium in a process believed to resist IAV infection [12].

Various NLRs are expressed by airway epithelial cells. NOD1 activation can reduce airway hyperresponsiveness and decrease allergen-specific T-cell proliferation in allergen-induced lung inflammation [13]; its expression is downregulated during pollen season among patients with allergic rhinitis [14]. NLRP3 mediates airway epithelial cell responses to inhaled particulate matter, for example, PM10 [15].

A major function of epithelial CLRs is to sense fungal species, for example, dectin-1, which recognises *β*-glucan motifs in *Aspergillus fumigatus* and house dust mite (HDM) [16] whereas nonfungal allergens, such as Derp1 and cockroach allergen, which have proteolytic properties, can elicit allergic airway inflammation via PAR-2. Bitter and sweet taste receptors (T2R and T1R, resp.) are G-protein-coupled receptors expressed in respiratory epithelia (reviewed in [17]) that are activated by quorum-sensing molecules such as homoserine lactones from *Pseudomonas aeruginosa* or sugars. Their activation can enhance mucociliary clearance [18]. In addition, new roles are emerging for other receptors expressed by airway epithelium such as fractalkine receptor CX3CR1 [19] and the short-chain fatty acid receptor GPR41 [20].

In addition to membrane-bound PRRs, in the lung, soluble forms also exist that include mannose-binding lectin (MBL) and surfactant proteins A and D. Ligands for MBL include high mannose and N-acetylglucosamine oligosaccharides that trigger the activation of the classical complement pathway [21]. Surfactant proteins (SP) B and C lower alveolar surface tension whereas SP-A and SP-D bind to macrophages and stimulate their chemotaxis [22] or Gram-negative bacteria and inhibit T-cell proliferation [23], respectively.

### 2.4. Innate Immunity

The airway epithelium plays a key role in the lung's innate immune responses. Together with the complement system, the antiproteases, antimicrobial peptides, and cytokines expressed by airway epithelial cells or present within the airway lumen represent major factors that function to rid the lungs of infectious or invading microbes and cope with toxic intrapulmonary insults due to pathogens or environmental pollutants.

The complement system opsonizes pathogens and generates chemotactic peptides and the membrane attack complex [24]. Alpha-1 antitrypsin (AAT), secretory leukoprotease inhibitor (SLPI) and elafin are the most abundant serine antiproteases in the lung. Each has important beneficial effects within the lung and their dysregulated activity can adversely impact on the inflammatory process (Figure 2(b)) [25–30].

Antimicrobial peptides form part of the lung's innate immune defences, and inactivation of these peptides has been implicated in airway infections (Figure 2(b)). Lactoferrin is a monomeric iron-binding glycoprotein (76–80 kDa) present in the secondary granules of neutrophils [31]. It plays a microbiostatic role in mucosal fluids [32] and also has antiviral, antifungal, and anticancer activities and can act as an immunomodulator. The primary functions of LL-37 are to eliminate pathogens, inhibit biofilm, and enhance the immune responses [33]. Human beta-defensins are small (4–6 kDa) cationic peptides. Twenty-six human *DEFB* genes have been identified but only HBD1–4 are secreted by the respiratory tract and play a role in airway mucosal defense. HBDs possess antibacterial [34], antiviral [35], and antifungal properties [36]. HBD-2 and HBD-3 also have immunomodulatory properties [37].

## 3. Mucus Production/ASL Regulation in CF and COPD

**Figure 3:**
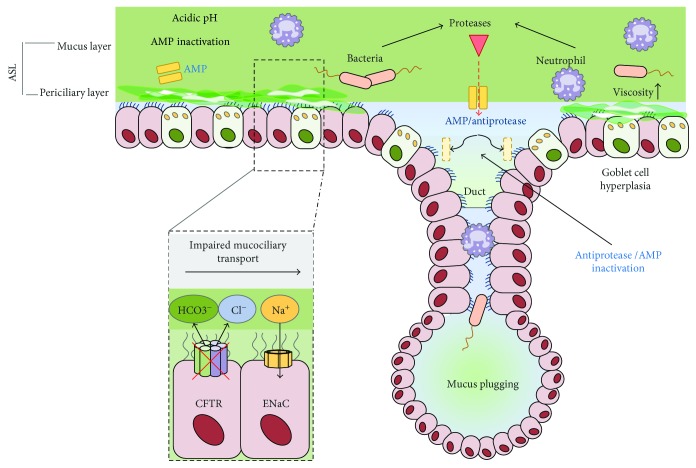
Pathophysiology of airway disease in cystic fibrosis. In cystic fibrosis, loss of normal CFTR function and overactivity of ENaC result in acidification (loss of HCO3^−^ secretion) and dehydration of the airway surface liquid layer (ASL) disrupting the normal mucociliary escalator. This results in an increase in ASL viscosity, mucus plugging, bacterial colonization, and neutrophil-dominated inflammation. An overabundance of bacterial and neutrophil derived proteases degrades important antiproteases and antimicrobial peptides in the CF airways further compounding an already-overwhelmed impaired innate immune system.

As previously described, the airway epithelium is covered by the ASL that comprises a mucus layer and the underlying PCL. Airway mucus is responsible for hydration of the epithelium and acts as an essential protective component of the innate defense system of the airways [38, 39]: inhaled microorganisms, noxious agents, and particles get entrapped in mucus and eliminated by mucociliary clearance (MCC) and/or cough, thus protecting the lung from airway infection and inflammation. The two main secreted airway mucins are MUC5AC and MUC5B [38, 40]; the latter but not MUC5AC seems to be required for airway defence against bacteria [41]. Properly hydrated mucus is a critical component of airway homeostasis; failure to maintain adequate mucus hydration, mucus hypersecretion, and altered mucus properties lead to impairment of mucociliary clearance and are prominent features of chronic airway diseases such as CF and COPD, contributing to airway obstruction in these diseases. Ion channels that regulate Na^+^ and Cl^−^ transport are required for the proper hydration and composition of ASL. CFTR is the primary apical ion channel involved in Cl^−^ transport in the lung epithelium; in addition, it is also implicated in the regulation of ENaC that is rate limiting for Na^+^ and fluid absorption from the airway surface. Additional ion channels in the airway epithelium play a role in maintaining ASL homeostasis, including the solute carrier 26A (SLC26A) family of anion exchangers and calcium-activated chloride channels [42, 43]. Alterations in ASL composition and disruption of this tightly regulated airway milieu have been linked to the pathogenesis of CF lung disease and COPD. In CF, the defect/dysfunction of the CFTR protein causes a defect in chloride and bicarbonate transport into the airway lumen that results in dehydration and acidification of ASL, production of viscous, acidic, and altered secretions, with impairment of MCC; this in turn induces airway obstruction, favouring chronic bacterial infection and inflammation (Figure 3). Recent studies showed that the ASL volume depletion causes a collapse of cilia and impairs mucus transport, further supporting the concept that ASL dehydration is a crucial mechanism in CF lung disease [42]. The role of airway surface dehydration as a disease-initiating mechanism is also supported by studies in *β*ENaC transgenic mice and in SCL26A9 mice [44–48]. Recent biophysical studies provided novel insight on the effects of airway surface dehydration, showing that this leads to an increase of the concentration of secreted mucins and consequently of the osmotic pressure of the mucus layer; when the latter increases above a critical threshold, the PCL and the cilia get compressed leading to stasis and adhesion of mucus on the airway surface [49]. This critical mucus hyperconcentration may be induced not only by the CFTR ion transport defect but also by airway inflammation; thus, it may play an important role in the pathogenesis of chronic inflammatory diseases such as COPD [50].

In addition to airway surface dehydration, the impaired bicarbonate secretion mediated by CFTR dysfunction results in altered mucus structure, increased viscosity and abnormal mucus secretion; therefore, it may contribute to mucus plugging and the impairment of MCC in CF lung disease beyond that caused by airway dehydration alone. Several recent studies support the key role of bicarbonate secretion in regulating local pH and the airway milieu and in maintaining normal mucus properties: bicarbonate, in fact, drives ionic content and fluids on epithelial surfaces and allows mucins to unfold, and its depletion has been shown to result in dense mucus and increased ASL viscosity thus impairing mucociliary transport [51–53]. Altered ASL viscosity would be a primary CF defect that might contribute, at least in part, to the pathogenesis of CF lung disease. Subsequently, infection, inflammation, and airway remodelling, with their consequences, may further modify ASL viscosity and further enhance the defect of mucociliary transport. Loss of CFTR-mediated bicarbonate secretion has also been shown to inhibit the activity of antimicrobial peptides present in ASL [53]. Thus, dysfunction of CFTR and the subsequent lack of bicarbonate and chloride secretion create an abnormal airway milieu, with impaired innate host defences that favours chronic airway infection and inflammation leading to progressive lung injury and ultimately to respiratory failure (Figure 3). Furthermore, recently, it has been shown that the loss of CFTR leaves H^+^ secretion by the nongastric H^+^/K^+^ adenosine triphosphatase (ATP12A) unchecked, which further decreases the ASL pH further impairing airway innate host defences [53].

Cigarette smoking is the major risk factor for COPD, a disease that shares some phenotypical features with CF, such as impaired mucociliary clearance, chronic airway inflammation, progressive airflow obstruction, and recurrent bacterial exacerbations. In particular, COPD patients with the chronic bronchitis phenotype exhibit pathologic and clinical features similar to CF, including goblet cell hyperplasia, mucin hyperexpression, and mucus accumulation and hypersecretion that contribute to bacterial infection and subsequent inflammatory responses and have been associated with lung function decline in COPD patients (Figure 1).

**Figure 4:**
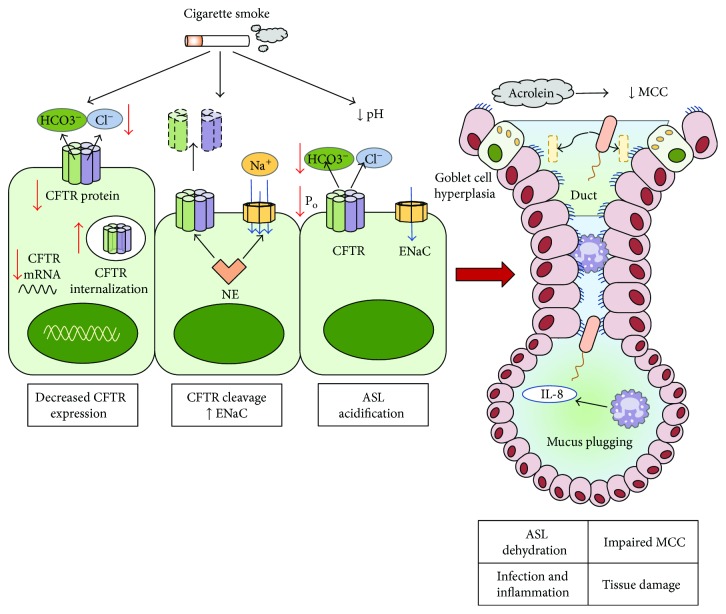
Effects of cigarette smoke on CFTR and pathophysiology of airway disease in COPD. Cigarette smoke components (e.g., acrolein and cadmium) can decrease expression and function of the CFTR protein by decreasing CFTR mRNA and protein levels, accelerated CFTR internalization, and decreased channel opening probability (*P*_o_). Increased levels of neutrophil elastase in COPD may worsen CFTR dysfunction by degrading CFTR and upregulating ENaC expression. Loss of CFTR function results in ASL dehydration and acidification, mucus hypersecretion, and mucus plugging leading to reduced mucociliary clearance, chronic inflammation, impaired innate immunity, and infection.

Increasing evidence shows that cigarette smoking induces an acquired CFTR dysfunction in patients with normal CFTR gene. Welsh firstly reported that cigarette smoke decreases chloride secretion in the airway epithelium [54]. Several subsequent studies then showed that sustained exposure to cigarette smoke and cigarette smoke extract (CSE) was able to reduce CFTR expression and function *in vivo* in smokers and in COPD patients and in airway epithelial cells *in vitro* [55–58]. Several independent mechanisms have been proposed to account for these effects of smoke, including aberrant transcript expression, direct effects of smoke metabolites on CFTR function, and accelerated internalization of CFTR Cl^−^ channels from the apical plasma membrane (Figure 4) [55, 56, 58–61]. Smoke-induced CFTR dysfunction results in ASL dehydration, and it was shown that ASL height decreased permanently after chronic smoke exposure due to changes in active ion transport, indicating an effect of smoke on ASL homeostasis. It has also been shown that CSE is capable of inducing a delayed mucociliary transport *in vitro* and a marked increase of mucus expression both *in vitro* and *in vivo*. CSE has also been shown to be capable of inducing MUC5AC expression and mucus hyperproduction in AEC, an effect that seems to be mediated through ROS-dependent autophagy [62–64]. Together with mucin hypersecretion, smoke-induced CFTR dysfunction will aggravate mucus hyperconcentration and plugging in COPD airways (Figure 4) [42, 65–68]. Tobacco smoke components such as acrolein and acetaldehyde have been shown to impair mucociliary clearance [69]; furthermore, acrolein and cadmium inhibit CFTR function *in vitro* and can reach detectable levels in humans as well (Figure 4) [70, 71].

In ciliated cells from nasal brushing of smoke-exposed subjects and moderate/severe COPD patients, ciliary beat frequency was significantly reduced compared to control subjects [72, 73]. Furthermore, CS exposure has been shown to affect ciliogenesis and to cause ciliary shortening [74], effects that further contribute to the impairment of MCC.

Reduced MCC leads to accumulation and adhesion of mucus in the airways, favouring retention of irritants and noxious agents contained in cigarette smoke. This in turn triggers exaggerated inflammatory/immune responses, with recruitment of neutrophils and macrophages and excess of protease release that promotes the formation of emphysema in the lungs of smokers with COPD [60, 75]. Recent studies shows that CFTR protein expression correlates inversely with emphysema severity in lungs of COPD patients, suggesting that impaired CFTR function may also be implicated in emphysema formation in humans [76]. A role of CFTR dysfunction in the pathogenesis of emphysema is further supported by studies in *β*ENaC transgenic mice demonstrating that CF-like airway surface dehydration does cause not only chronic mucus obstruction and airway inflammation but also emphysematous changes in these mice [44, 46, 77]. Interestingly, we demonstrated that genetic deletion and pharmacologic inhibition of PI3K*γ* decreases both neutrophilic airway inflammation and structural lung damage in *β*ENaC transgenic mice [78].

As previously discussed, defective CFTR also decrease bicarbonate secretion, that is, crucial in pH ASL regulation and in maintaining normal mucus properties. Tobacco smoke exposure decreases bicarbonate secretion presumably resulting in acidification of ASL and increased ASL viscosity (Figure 4) [42, 79, 80].

A marked decrease of CFTR function has been reported in the upper and lower airways of smokers and patients with COPD; interestingly, in COPD patients, significant correlations have been shown between clinical manifestations such as dyspnea and symptoms of chronic bronchitis and the levels of CFTR suppression in their lower airways [56]. The marked oxidant burden and neutrophilic inflammation associated with COPD and particularly with advanced disease further aggravate the effects of CFTR dysfunction in COPD airways. Studies using cell lines expressing wild-type CFTR have shown that a prolonged exposure to oxidant stress with either t-butylhydroquinone or cigarette smoke extract was capable of inhibiting CFTR function, protein levels, and gene expression [55, 81, 82]. Furthermore, it has been reported that CS-induced CFTR dysfunction and the associated inflammatory/oxidative stress cause an impairment of autophagy that also contributes to the development of emphysema [56, 82, 83].

All these data support the concept that CFTR dysfunction caused by cigarette smoke may contribute to the pathophysiology of COPD and may represent a potential target for the development of novel therapeutic approaches.

## 4. Innate Immune Response in CF and COPD Epithelium

The airway epithelium contributes to host defence by a variety of mechanisms including its barrier function and mucociliary clearance, as well as by the production of antimicrobial peptides and proteins and a range of cytokines, chemokines, and growth factors that mediate leukocyte recruitment, modulation of innate and adaptive immunity, and tissue repair and remodelling [84–86]. Through these mechanisms, the airway epithelium contributes directly to host defence and augments the immune response via the recruitment of inflammatory/immune cells and the interaction with other structural cells in the airway wall.

### 4.1. Innate Immune Responses in CF Epithelium

A vicious cycle of infection followed by intense neutrophil-dominated inflammation, ineffective clearance of infection, and irreversible airway destruction is characteristic of CF. Airway inflammation is ultimately driven by a dysfunctional epithelium due to an impairment in innate host defence systems, secondary to mutations of CFTR [87–90]. Defective bicarbonate secretion due to CFTR dysfunction reduces ASL pH, impairing the activity of antimicrobial peptides and mucus properties. Despite the ineffectiveness of the CF airway surface to remove mucus, goblet cells are likely to continue secreting mucins [91] leading to plugging. Specific defects in many aspects of the lung's innate immune repertoire are evident in CF.

The CF airway is a TLR agonist-rich environment, and chronic inflammation in CF may for the most part be due to activation of TLRs [92]. Although TLR hyperresponsiveness is largely accepted to contribute to CF lung disease, hyporesponsiveness to TLRs can also negatively impact on pulmonary inflammation. During high circulating oestrogen (E2) states in the female CF airways, a TLR hyporesponsiveness, manifested by reduced IL-8 release, occurs in response to a range of bacterial agonists [93]. A hyporesponsive state may be problematic, leading to ineffective clearing of microorganisms.

NOD1 (or CARD4) and NOD2 (or CARD 15) detect peptidoglycan of Gram-positive or Gram-negative bacteria, and their activation causes induction of proinflammatory NF-*κ*B-dependent cytokines [94]. IL-17 has been shown to augment expression of NOD1, NOD2, and TLR4. By upregulating these PRRs, IL-17 primes the airway to increase its inflammatory response (IL-8) to bacterial ligands. Interestingly, pretreatment of CFTRΔF508 cells with IL-17 resulted in a 10-fold increase in IL-8 synthesis following treatment with a NOD1 agonist, highlighting the importance of NOD1 as contributor to aberrant inflammatory responses in the CF lung [95].

Polymorphisms in the mannose-binding lectin 2 (MBL2) promoter can affect MBL serum levels. Relative deficiency of MBL appears to accelerate the age-related decline in lung function in CF patients as MBL-deficient patients older than 15 years of age displayed poorer lung function compared with those younger than 15 years of age [96].

Likewise, relative reduction in SP-A and SP-D has been observed in patients with CF [97] and levels inversely correlate with inflammation and bacterial burden [98]. The cause may be related to the ability of *P. aeruginosa* elastase and protease IV to degrade SP-A and SP-D in the CF lung [99, 100]. Furthermore, CFTR-dependent alterations in complement-mediated interactions between *P. aeruginosa* and monocytes may contribute to enhanced susceptibility to infection in patients with CF [101].

An inherent proinflammatory state exists in the CF airways and may precede bacterial colonization. Overproduction of the neutrophil chemokine IL-8 by CF airway epithelial cells may be a consequence of both intrinsic CFTR dysfunction [102] and infection [103]. Elevated concentrations of proinflammatory cytokines including TNF-*α*, IL-1, and IL-8 have been found in children with CF [104]. TNF-*α* concentrations in sputum are not significantly correlated with clinical status but do show a strong correlation with sputum IL-8 levels [105].

Interleukin 17 (IL-17-A/F) is a proinflammatory cytokine with roles in the immunopathogenesis of CF and COPD. Elevated IL-17A mRNA and protein in CF sputum implicate this cytokine in the persistent neutrophilic infiltration in CF lung disease [106]. IL-17 positively regulates the production of proneutrophilic mediators from CF epithelial cells by increasing IL-8 and IL-6 [107]. IL-17A is significantly higher in BALF of symptomatic patients as compared with clinically asymptomatic patients with CF, and its increased concentrations precede infection with microbes such as *P. aeruginosa* and thus have clinical relevance [108].

Antiproteases within the CF lung are susceptible to cleavage and inactivation by host proteases (Figure 2(b)). SLPI is susceptible to cleavage by cathepsins B, L, and S [109] and elafin by NE in lungs of patients colonized with *P. aeruginosa* [110]. Although levels of AAT rise in acute inflammation, in the CF lung, the inhibitory activity of AAT is overwhelmed by an excess of NE.

While lactoferrin has been reported to be present at higher concentrations in the CF lung compared with normal lungs [111], its activity may be suboptimal secondary to cleavage by *Pseudomonas* elastase and NE [112]. Cleavage of lactoferrin by cathepsins B, L, and S also results in loss of its microbicidal and antibiofilm activity, and in the CF lung, this may potentiate the biofilm forming ability of *P. aeruginosa* [113].

The effectiveness of LL-37 may be hampered in the CF lung through complexing with polymers including GAGs or DNA released from the breakdown of neutrophils and other cells [114, 115].

Finally, variant alleles (single-nucleotide polymorphisms) in the DEFB1 gene encoding HBD-1 may contribute to the colonization of *P. aeruginosa* in CF [116]. Beyond that, cysteinyl cathepsins which are present at higher than normal levels in the CF lung cleave and inactivate HBD2 and HBD3 [117].

### 4.2. Innate Immune Responses in COPD Epithelium

An increasing number of studies demonstrate that epithelial defence functions are altered or decreased in chronic airway diseases including COPD and that, as described in CF, defects in the innate immune system play a relevant role also in the pathogenesis and pathophysiology of COPD [5, 42, 118–123]. Cigarette smoke induces relevant alterations in the airway epithelial architecture and impairs epithelial barrier function by increasing the permeability of the airway epithelium, decreasing ciliary function, and reducing mucociliary clearance. This epithelial barrier dysfunction in turn can increase the entry of pathogens and noxious particles into the airway mucosa, further impairing the barrier function and host defences [4, 5, 39, 124, 125–130]. Cigarette smoke induces mucous cell hyperplasia and mucus hypersecretion contributing to airway obstruction; in the small airways, smoking-induced goblet cell hyperplasia is associated with loss of club cells, responsible for the production of secretoglobin, surfactant protein, and other defense factors [131]. A reduced expression of polymeric immunoglobulin receptor/secretory component (pIgR/SC) has also been reported in the COPD epithelium that impairs the transepithelial transport of secretory IgA and correlates with the severity of airflow obstruction [132]. Cigarette smoke may affect epithelial cell functions through a variety of mechanisms, including direct oxidant activity, TLR signalling, ER stress induction, and activation of the integrated stress response [133, 134]. In addition to impairing barrier function, cigarette smoke exposure increases the release of inflammatory mediators by AEC, whereas it decreases the expression and activity of ASL antimicrobial peptides (AMP). This defect, caused by smoking-induced CFTR dysfunction, is common to CF airways and contributes to the increased susceptibility to respiratory infections shared by both diseases. The antimicrobial activity of AMPs may also be affected by other conditions occurring in both CF and COPD airways such as AMP degradation by microbial and host proteases and AMP inhibition by microbial polysaccharides, F-actin, and DNA from dying cells. Cigarette smoke also inhibits the molecular pathways involved in interferon production in response to viral infections [135]. Whereas the expression and activity of epithelial AMP are impaired in COPD, the levels of neutrophil-derived peptides and LL-37 are increased as a consequence of the marked neutrophilic inflammation in this disease and further increase during disease exacerbations [136–145]. Likewise, increased levels of lysozyme and lactoferrin have been reported in BALF of patients with COPD [121, 146], whereas SLPI levels were reported to be increased in stable patients, but decreased during disease exacerbations [144, 147, 148]. However, some inconsistent findings concerning sputum AMP levels in COPD patients have been reported in previous studies [136–139, 149] that may be due to the small sample sizes of these studies and the different clinical conditions and disease stage of patients.

Exposure to cigarette smoke also leads to activation of several PRRs, either directly by components of smoke or indirectly by causing injury to epithelial cells [84, 121, 134, 150, 151]. As in CF, also in COPD, TLR4 has been suggested to play a key role in the inflammatory/immune response: TLR4-defective mice show attenuated lung inflammation after challenge with cigarette smoke [152–154]. Elevated levels of damage-associated molecular patterns (DAMPs), such as high-mobility group box 1 (HMGB1) [155], uric acid, and extracellular ATP [156], have been observed in the BALF of patients with COPD compared with smokers without COPD. Extracellular ATP has been shown to activate the NLRP3 inflammasome, regulating the expression of interleukin-1*β* and IL-18, [153] particularly during disease exacerbations [157, 158]. In IL-1R knock-out mice, pulmonary inflammation induced by acute exposure to cigarette smoke was attenuated and these mice were protected against emphysema after chronic smoke exposure [159].

Activation of AEC by cigarette smoke or pathogens also triggers an inflammatory response, with the release of cytokines and chemokines such as TNF-*α*, IL-1, IL-6, GM-CSF, and IL-8 acting on inflammatory/immune cells as well as on resident cells [5, 84, 85, 119, 121, 134, 150, 152, 158].

Similar to what occurs in CF airways, activated AEC from COPD patients release more IL-8 than cells from smoking control subjects [160] and display a proinflammatory phenotype in culture [161], although the molecular mechanisms are different in CF and COPD. Levels of IL-17 and other Th17 cytokines are also increased in sputum and airways of patients with COPD as reported in CF, further enhancing neutrophil recruitment. [162, 163]. Whereas physiologically the inflammatory process induces protective immune responses, in patients with COPD and CF, chronic airway inflammation amplifies the tissue damage and further impairs local immune defences, thus contributing to susceptibility to recurrent infections. Neutrophils and alveolar macrophages are increased and activated in COPD, as well as in the CF lung, and contribute to the oxidant burden and the protease/antiprotease imbalance that drive the development of emphysematous changes in both diseases. Furthermore, neutrophils from COPD patients show altered chemotactic responses [164] that impair their defence function whereas macrophages show reduced phagocytic ability [165–167] and impaired innate responses to respiratory pathogens. These alterations facilitate the chronic colonization of the airways and infectious exacerbations [168]. An increased number and activation of dendritic cells in the lung of patients with COPD have also been reported in some studies that seem to correlate with disease severity [169–171], whereas other studies have found decreased numbers of these cells and an impaired maturation status [172–175]. Recent studies suggest that innate lymphoid cells (ILC), with important roles in immune homeostasis and lung immunity, may play a role in COPD [176–178]. In mice exposed to cigarette smoke, a strong type 1 response to influenza virus was observed that was associated with a transdifferentiation of ILC2 into ILC1 cells, induced by IL-12 and IL-18 [178]; in patients with COPD, an increase of ILC1 and a decrease of ILC2 cells was reported in lung tissue [178, 179]. Finally, recently, it has also been reported that pulmonary natural cytotoxicity receptor- (NCR-) ILC3 cells tend to accumulate into the lungs of COPD patients [177]; these cells produce IL-17A and IL-22 and might contribute to driving neutrophilic inflammation in the COPD airways. Interestingly, these cytokines are also crucial in the formation of lymphoid follicles [180, 181], the numbers of which are increased around the small airways and in the lung parenchyma [182–184] in severe COPD as well as in peribronchial, parenchymal, and perivascular areas in the lung of CF patients with advanced lung disease [185]. The peribronchial localization of tertiary lymphoid organs and the epithelial expression of IL17A and chemokines involved in their development suggest a role for airway epithelium in lymphoid neogenesis in these diseases. However, differently from COPD, a shift from B cell to T-cell predominance has been observed in CF lymphoid follicles, suggesting that the cellular adaptive immune response is specifically affected in CF [185]. Recently, Frija-Masson and colleagues [186] showed that peribronchial lymphoid neogenesis was induced in the lungs of mice upon persistent bacterial infection, suggesting that chronic bacterial infection contributes to the lymphoid neogenesis observed in both COPD and CF.

A growing body of evidence now suggests that dysfunction of epithelial innate immune responses and the consequent chronic airway inflammation can drive the initiation, exacerbation, and progression of chronic inflammatory disease such as COPD and CF. In this context, therapeutic targeting of dysfunctional immune responses could be an interesting strategy in the treatment of these diseases.

## 5. Comparison of Epithelial Remodelling in CF and COPD

Efficient epithelial regeneration following injury is crucial in tissue homeostasis and prevention of disease. Exposure to repeated noxious/inflammatory stimuli as well as airway epithelial dysfunction affects epithelial regeneration and repair pathways and the ability of epithelial cells to restore barrier functions; this leads to aberrant remodelling and structural damage that further impair epithelial functions and generate a vicious cycle favouring aggressions by potential exogenous insults.

Both CF and COPD are characterized by dysfunctional airway epithelial repair and remodelling that impair lung architecture and contribute to disease pathogenesis and progression.

In CF, progressive remodelling of the airways ultimately results in structural damage with development of bronchiectasis, emphysema, and impaired lung function. Differently from COPD, structural changes of the airway and alveolar wall in CF appear early in life. There is still debate about the sequence of events leading to remodelling and structural alterations in CF and their relationship to infection and inflammation; in particular, it is still debated whether structural changes are related to and initiated by infection/inflammation or are a result of CFTR dysfunction independent of infection and inflammation [87–90, 102–104]. Hyperplasia of goblet cells and basal cells [187–190], squamous metaplasia [187, 190, 191], increased epithelial height [187, 192], cell shedding [187–190, 193], with loss of ciliated epithelial cells, and a disorganization of tight junctions and compound cilia [192, 194–196] have been reported in the context of epithelial remodelling in CF airways; extensive structural changes of the small airway epithelia have also been observed, including epithelial shedding and altered barrier integrity [197]. Conflicting results have been reported concerning epithelial and reticular basement membrane (RBM) in adult patients that was found thickened in some studies [189, 198, 199], but significantly thinner than normal in other studies [200]. Interestingly, many of these structural changes are similar to those observed in COPD, although the molecular mechanisms initiating these changes are different.

Whereas until recently, little was known about the frequency of occurrence and the clinical relevance of emphysematous changes in CF lung disease, recent studies show that, with the remarkable increase of patient survival, emphysema is now a more prominent disease component in CF. Using quantitative CT measurements, Wielpütz and colleagues [201] showed that early onset and progressive emphysema is a characteristic feature of CF lung disease; emphysema is observed in early adolescence, increases in adult CF patients, correlates with lung function, and contributes to disease severity. Mets and colleagues [202] pathologically and radiologically confirmed that emphysema is common in advanced CF lung disease, is related to age, and in some cases approaches the changes observed in explanted lungs of COPD patients. Thus, emphysema might become an increasingly important disease component in the aging CF population. Several mechanisms mainly related to CFTR dysfunction may induce emphysema formation in CF patients; among these, chronic inflammation with protease/antiprotease imbalance and extracellular matrix proteolysis, altered ceramide metabolism, alveolar apoptosis, and defective autophagy [63, 76, 203]. Most of these mechanisms are common to those involved in the pathogenesis of smoke-induced emphysema.

Some studies have reported that CFTR plays an important role in regulating the early events of epithelial cell migration [188, 198, 200] and that CF airway epithelial cells exhibit slower migration and wound repair than non-CF cells [188, 198, 204]. The findings that the pharmacological rescue of CFTR function in CF cells significantly improved wound healing and that inhibition of CFTR expression or activity decreased proliferation and migration of non-CF airway epithelial cells [188, 198] further support the role of CFTR in modulating airway epithelial cell migration and the concept of a dysfunctional airway epithelium in CF. Data obtained on tissues from CF fetuses (taken at autopsy) or in a humanized nude mouse xenograft model and in CF mice suggest that the epithelial remodelling starts to occur prenatally and in the absence of infection [205–207]. A recent *in vitro* study, using CF human airway epithelial cell cultures, confirmed and extended these findings, showing that CF epithelium regeneration, even in the absence of exogenous infection/inflammation, was abnormal and associated with basal cell hyperplasia and with delayed ciliated cell differentiation. The findings of this study demonstrate that the abnormal remodelling in CF epithelium was partly induced by the intrinsic hyperinflammatory phenotype of CF cells [208]. Interestingly, in this study, an increase in CF epithelium height was observed in the context of epithelium remodelling. An increased epithelium height was previously reported *in vivo* in CF lungs as compared with lungs from COPD patients [192] and in these latter patients as compared with non-COPD subjects at both the nasal and bronchial levels [209]. Similarly, basal cell hyperplasia, a finding associated with increased epithelium height, was also reported *in vivo* in CF [187, 189] and COPD lungs [210]. It has been already emphasized that chronic inflammation is a hallmark of CF; inflammation begins early in the disease even in the apparent absence of infection, and it has been postulated that it may be dysregulated in the CF lung as a consequence of defective CFTR [87–90, 102–104, 211, 212]. Interestingly, stimulation of non-CF airway epithelial cells with proinflammatory cytokines mimicked the abnormal remodelling observed in CF epithelial cells and induced an increase of epithelium height and basal cell numbers comparable to those observed in CF cultures [208]. Overall, the findings of the previous studies suggest that exogenous infection and inflammation are not the exclusive factors affecting airway epithelium remodelling in CF and that the intrinsic dysfunction of CF airway epithelial cells plays an important role in this process and in the alterations of epithelial regeneration following injury. A growing number of *in vitro* and *in vivo* studies support the notion that chronic neutrophilic inflammation is a key factor in structural lung damage and lung function decline in CF through the release of damaging neutrophil products such as NE and reactive oxidant species [211, 212]. Airway inflammation is further augmented after onset of chronic airway infection. A vicious cycle of neutrophilic inflammation, noxious mediator release, and overwhelmed defences occurs within the airways that further exacerbates epithelial injury and remodelling, leading to disease progression and irreversible lung damage. The chronic neutrophilic inflammation, heightened during acute exacerbations of the disease, is a common process in CF and COPD and may be involved in the common abnormalities observed *in vivo* in remodelled airway epithelium, as well as in the destructive alterations of airway and alveolar walls.

There is evidence of degradation of structural components of the extracellular matrix (ECM) such as elastin, collagen, and glycosaminoglycans in the CF lung; these alterations are associated with the marked and early protease/antiprotease imbalance, with the release of high quantities of matrix metalloproteases (MMP), NE, and other proteases that contribute to the tissue damage and development of bronchiectasis and emphysema [213–216]. There is increasing evidence that the high levels of free NE activity observed in sputum and BALF from CF patients not only are able to cleave a number of ECM proteins but have also other direct and indirect deleterious effects on the epithelial repair process: in fact, they significantly impact the adhesion, proliferation, and wound repair of primary AEC [217]. There is also considerable *in vivo* evidence of an imbalance of MMP and their inhibitors (tissue inhibitors of MMP, TIMPs) in the CF airways with prevalence and activation of MMP [214, 218–221]. Activation of MMP-7 has been shown to inactivate AAT, thus further augmenting the effect of free NE activity [221], whereas the release and activation of MMP12 is involved in alveolar wall destruction [63]. A recent proteomic study of CF AEC showed significantly increased secretion of ECM proteins fibronectin, laminin, perlecan, and galectin-3 [222], whereas the CFTR defect was associated with a decreased integrin *β*1 signaling and a reduced wound repair [223]. These studies further support a role of mutant CFTR protein in the abnormal migration and reparative properties of the CF epithelium. Fibrotic changes associated with small airway narrowing have also been described, in particular in end-stage CF [224–226]. A recent study, using micro CT and histology documented dilatation and obstruction of distal airways and a severe reduction in the number of functional terminal bronchioles in end-stage CF lungs, confirming that, similar to COPD, obstruction and remodelling of peripheral airways are a prominent feature in CF lung disease [226].

Similar to CF, the airway epithelium represents a primary site of relevant molecular and morphologic changes in COPD; structural modifications of the epithelium in COPD resemble that observed in CF and include basal cell hyperplasia, squamous metaplasia, goblet cell hyperplasia, decreased integrity of the apical junctional barrier, and epithelial shedding [132, 222, 227]. Moreover, a reduction of cilia length and ciliary beating have been observed in COPD and upon cigarette smoke exposure [72, 127]; altered function and/or number of ciliated and goblet cells impair mucociliary clearance, contributing to the development of airway obstruction similar to what occurs in CF. Although pathologic changes in COPD lungs involve both the airways and parenchyma, studies by Hogg and colleagues clearly showed that smoking-induced changes in the small airways are crucial in the development of airway obstruction in COPD and precede destruction of the alveolar structure [228], similar to what was observed in CF. Interestingly, recently, it has been reported that smoking is able to induce a distal-to-proximal reprogramming of the small airway epithelium in COPD lungs, with a shift toward the proximal airway epithelial phenotype. These changes represent a novel feature of small airway pathology in COPD and seem to be mediated by exaggerated epidermal growth factor/epidermal growth factor receptor signaling in small airway epithelium basal cells [229]. An increased expression of epithelial growth factor receptors has been described in AEC of COPD patients, which might contribute to squamous metaplasia and an increased risk of bronchial carcinoma [230].

Normal differentiation of epithelial cells in COPD is altered both in terms of cellular functions and of the polarity of the epithelial cells, notably through epithelial to mesenchymal transition (EMT). This leads to dysfunctional physical, chemical, and immune barrier functions; whilst aberrant repair and remodelling of the COPD epithelium further impair epithelial functions. Cigarette smoke increases epithelial permeability by disassembly of tight junction proteins via the epidermal growth factor receptor pathway [124]. This may favour viral, fungal, and bacterial translocation through the epithelium.

As described for CF AEC, Perotin et al. recently showed that wound repair is delayed in primary cultures of severe and very severe COPD [231]. The epithelial cells undergoing EMT lose their epithelial phenotype, become able to migrate along and through the basement membrane, and presumably participate to peribronchial fibrosis [232]. EMT is reported both in upper airways (in chronic sinusitis) [233, 234] and in small and large lower airways in COPD [235] and is induced by cigarette smoke and by TGF-*β* [236, 237]. Cigarette smoke also promotes Wnt signaling: Heijink and colleagues reported that cigarette smoke increases Wnt-5B expression in COPD AEC cultures and favours a mesenchymal phenotype through a mechanism involving TGF-*β*/Smad-3 [238]. More recently, Baarsma and colleagues showed an enhanced expression of noncanonical Wnt-5A in experimental models of COPD and in human COPD that functionally impaired canonical Wnt signal-driven alveolar epithelial cell repair [239]. Overall, these findings suggest that alterations in Wnt signalling may contribute to abnormal repair and remodelling in COPD. As already highlighted, a reduced expression of the epithelial pIgR has also been reported in COPD that impairs the transepithelial transport of secretory IgA [132, 240, 241]. Interestingly, a recent study shows that pIgR-deficient mice develop COPD-like airway and parenchymal remodelling, resulting from persistent activation of inflammatory signalling by an altered lung microbiome, thus suggesting that S-IgA deficiency may play a role in small airway remodelling and disease progression in COPD [242] These findings are consistent with the results of previous studies showing that reduced pIgR expression in COPD epithelium correlates with the severity of airflow obstruction in this disease [241].

The dedifferentiation of the COPD epithelium through aberrant regenerating mechanisms is responsible for an altered physical, chemical, and immune response to inhaled particles and microbes. Furthermore, epithelial cell activation by cigarette smoke and other inhaled irritants in COPD induces the release of inflammatory mediators, further enhancing chronic airway inflammation. All these events can increase airway epithelial damage and promote an excess of extracellular matrix deposition and airway fibrosis, driving disease progression in COPD [158]. Restoration of the epithelial integrity by targeting signalling pathways involved in aberrant repair and remodelling is promising but will need further research [243, 244].

## 6. Conclusions and Therapeutic Perspectives

Cystic fibrosis is a genetic disease caused by mutations in the CFTR gene, whereas COPD is mainly caused by environmental factors—mostly cigarette smoking—on a genetically susceptible background [1–4]. Although the etiology and pathophysiology of these diseases are different, they share key phenotypical features such as chronic neutrophilic inflammation, progressive airflow obstruction, and recurrent infectious exacerbations, suggesting the possibility of common mechanisms. In particular, COPD patients with the chronic bronchitis phenotype exhibit pathologic and clinical features similar to CF, including goblet cell hyperplasia, mucin hyperexpression, mucus accumulation, and hypersecretion that contribute to bacterial infection and subsequent inflammatory responses, and have been associated with lung function decline in COPD patients. Growing evidence suggests that cigarette smoking induces an acquired CFTR dysfunction in patients with COPD, reducing the expression and/or function of the protein, and that this CFTR dysfunction is involved in most of the pathogenetic pathways common to both COPD and CF.

Airway epithelial cells are among the first sites of contact for pathogens and other noxious environmental irritants and play a critical role in maintaining normal airway functions and the homeostasis of the airway milieu. Relevant molecular and morphologic changes occur in the airway epithelium in both CF and COPD, similar in some aspects, and a growing body of evidence suggests that airway epithelial dysfunction is involved in disease initiation and progression in both diseases. Structural and functional abnormalities in both airway and alveolar epithelium have a relevant impact on alteration of host defences, immune/inflammatory responses, and the repair processes, leading to progressive lung damage and facilitating chronic and recurrent airway infections in both CF and COPD.

An increasing number of studies show that CFTR dysfunction affects several physiological processes relevant to airway epithelial cell functions, including chloride and bicarbonate transport, mucin secretion and MCC, host defences, and inflammatory/immune responses. Thus, CFTR dysfunction represents a potential target for the development of novel therapeutic approaches. An improved understanding of the common mechanisms affected by CFTR dysfunction in CF and COPD and the relevance of CFTR dysfunction in the different COPD phenotypes would provide new insights into disease pathogenesis and will be important for developing novel therapeutic strategies relevant to both diseases. Recently, innovative and specific therapies targeting the CFTR defect have been developed; in the context of the increasing evidence about the role of CFTR dysfunction in COPD, the possibility that therapies designed to correct the CFTR dysfunction may benefit not only CF patients but also patients with COPD is attractive and need further studies.
